# Hemiplegic Migraine as the Initial Presentation of Biopsy Positive Cerebral Autosomal Dominant Arteriopathy With Subcortical Infarcts and Leukoencephalopathy

**DOI:** 10.7759/cureus.2631

**Published:** 2018-05-15

**Authors:** Garrett Rossi, Siddesh Shambhu

**Affiliations:** 1 Psychiatry, Cooper University Hospital, Camden, USA; 2 Medicine, Atlantic Health System

**Keywords:** stroke, stroke, mri, cadasil, cerebral autosomal dominant atreriopathy, migraine

## Abstract

The diagnosis of cerebral autosomal dominant arteriopathy with subcortical infarcts and leukoencephalopathy (CADASIL) in adults can be challenging. Initially, this disease can mimic embolic cerebral infarction, multiple sclerosis, and other neurological diseases on imaging studies. CADASIL is the most common hereditary cerebral angiopathy which is inherited in an autosomal dominant fashion. There is a wide variety of clinical presentations including a migraine headache, mood disturbances, cognitive dysfunction, and recurrent subcortical cerebral infarctions. This case details the hospital course and diagnosis of a 41-year-old male who initially presented with symptoms consistent with his previous diagnosis of a hemiplegic migraine who was later found to have biopsy-positive CADASIL after the symptoms failed to remit.

## Introduction

Cerebral autosomal dominant arteriopathy with subcortical infarcts and leukoencephalopathy (CADASIL) is the most common cause of inherited stroke in adults. The acronym CADASIL was used to describe the features of the disease in the 1990s [1]. The condition is characterized by recurrent subcortical infarctions and vascular dementia. Other clinical features include changes in mood, cognitive dysfunction, and migraine. The incidence and prevalence in the United States is estimated at 1 to 5 per 100,000 individuals, but definitive values are unknown at this time [2]. The median survival time is estimated to be 64 years for males and 69 years for females, with the full spectrum of disabilities present by 45 years of age [3]. CADASIL is caused by a mutation in the NOTCH3 gene on chromosome 19q12 [4]. This gene codes for a transmembrane receptor protein and is located on the smooth muscle surrounding arteries. It is this mutation that leads to thickening and fibrosis of the small and medium-sized arteries which is responsible for the recurrent ischemic subcortical infarctions [1]. The classic presentation for CADASIL is migraine, psychiatric disturbance, dementia, and recurrent strokes [5]. Migraine with aura is a common symptom associated with CADASIL. It can be the initial symptom of the disease and occurs in approximately 30% of patients [3]. The pathway by which a mutation in the NOTCH3 gene results in increased migraine with aura is still unknown [4]. Hemiplegic migraine is also known as a complex migraine and is characterized by unilateral weakness and migraine headache. The primary feature that separates hemiplegic migraine from other types of migraine with aura is the presence of motor weakness as a manifestation of at least some attacks. The estimated prevalence of hemiplegic migraine is 0.01% [6]. The familial and sporadic forms occur with equal prevalence [6]. This case details the history of a 41-year-old male patient with an established diagnosis of hemiplegic migraine, who presented with right-sided facial droop, and motor weakness in the right lower extremity. The diagnostic work up included several possible etiologies for the patient’s symptoms. He was eventually diagnosed with CADASIL after a biopsy was taken during vasculitis work up.

## Case presentation

The patient was a 41-year-old gentleman with a past medical history of hepatitis C, hemiplegic migraine, and diverticulitis. He was well until 2011 when he first developed a severe headache followed by right-hand weakness and numbness. He was taken to the hospital where a diagnosis of hemipalegic migraine was made. Magnetic resonance imaging (MRI) revealed some white matter changes thought to be secondary to hemiplegic migraine at that time. He recovered complete neurological function with no residual deficits.

**Figure 1:**
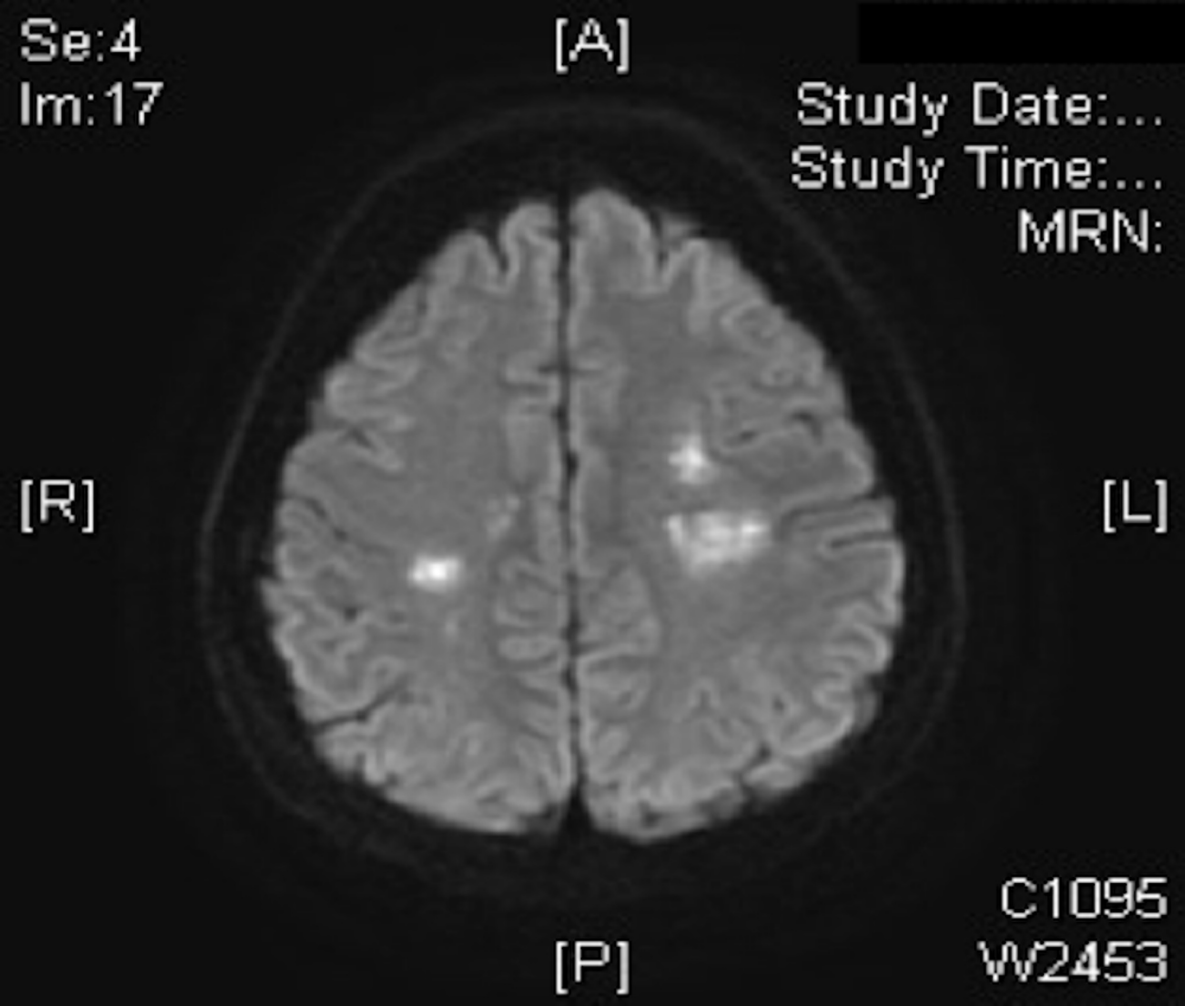
Multiple foci of restricted diffusion scattered bilaterally along the brain parenchyma involving multiple vascular territories

On this admission, the patient initially presented to the hospital with right-sided facial droop and weakness in the right lower extremity. The initial computed tomography (CT) scan of the brain was negative for acute bleed. An MRI of the brain was ordered because the patient continued to experience focal neurological deficits including right arm weakness, dysarthria, and decreased cognitive function on neurological examination and neuropsychiatric testing. At this point, it was thought that he had suffered an ischemic stroke, and the MRI showed multiple areas of restricted diffusion suggestive of embolic disease (Figure 1). These findings called into question the diagnosis of hemiplegic migraine.

The patient received aspirin after ruling out active bleeding on non-contrast CT of the head. The search for an embolic source included a CT-angiograph of the neck which did not reveal carotid stenosis. He then had a transthoracic echocardiograph and a trans esophageal echocardiograph both of which were unremarkable. The differential diagnosis at that time included transient ischemic attack, ischemic stroke, brain tumors, central nervous system (CNS) infection, CNS vasculitis, inherited disorders including mitochondrial encephalomyopathy, lactic acidosis, and stroke-like episodes (MELAS), CADASIL, and hereditary telangiectasia. A lumbar puncture was performed for possible meningitis. The cytology was negative and cerebrospinal fluid (CSF) analysis was not suggestive of meningitis, neurosyphilis, or Lyme disease. Lyme serology, the Venereal Disease Research Laboratory test (VDRL), and human immunodeficiency virus (HIV) blood tests were all negative. The CSF was also screened for possible multiple sclerosis, but no oligoclonal bands were detected. At this point, there was a concern for possible vasculitis as the cause of the patient's symptoms. Genetic testing for CADASIL was ordered, but the sample had to be sent out to a lab equipped to perform the genetic test and would take several weeks to return. He was placed on a five-day course of intravenous (IV) methylprednisolone which improved his neurological symptoms. Following completion of his steroid course, it was decided that an alternative to angiogram would be brain biopsy to look for CNS vasculitis. The patient and his family were eager to have an established diagnosis by the end of the hospital course, and the differential diagnosis included CNS vasculitis for which brain biopsy would provide valuable diagnostic information. Given the recent advances in imaging and neurosurgical techniques, the complication rate for routine biopsy has been reduced significantly. The patient elected to have the procedure performed. Rheumatology was consulted and an angiogram was recommended as well to rule out CNS vasculitis or vasospasm as a possible cause for the strokes. The patient had physical therapy and his motor and cognitive function gradually improved over the course of his hospital stay. The patient was accepted into a neurological rehabilitation facility and discharged on oral steroids. The results of the brain biopsy confirmed CADASIL after the patient was discharged to the rehabilitation facility. The results of the genetic tests for mutations in the NOTCH3 gene provided further confirmation with a sensitivity approaching 100%.

## Discussion

Migraine is one of the most common episodic headache disorders in which the pathophysiology of the symptoms is still largely undetermined [7]. At least part of the pathophysiology is explained in the classical vascular theory which states that the aura is the result of vasoconstriction and the headache is caused by vasodilation [8]. Newer theories suggest that migraine aura may be caused by cortical spreading depression, but a definitive mechanism is still unknown [9].

Motor aura is the hallmark of hemiplegic migraine, but it is not the only type of aura that can occur during an attack [10]. There are usually two or more aura symptoms experienced during an attack. Each attack tends to evolve over 20-30 minutes time, with the following symptoms appearing in order, visual aura, followed by sensory, motor, and brainstem symptoms [11]. Motor symptoms most often start in the hand, and gradually spread to the arm and face [12]. Hemiplegic migraine can mimic transient ischemic attack or stroke. With hemiplegic migraine, the aura symptoms tend to evolve over a longer period of time, and in a specific order. It is rare for the aura symptoms to develop acutely. In a minority of patient’s, motor weakness or other aura symptoms can develop acutely and may mimic a stroke. Most patient’s with hemiplegic migraine experience headache with each attack. The headache can be bilateral or unilateral, and with unilateral headaches, the headache may be ipsilateral or contralateral to the aura symptoms [11,13]. In between attacks, most patients have a normal neurological exam.

In most cases of hemiplegic migraine, imaging via CT or MRI are normal. In a small number of cases, some changes can be observed including cortical edema, and cortical and meningeal enhancement contralateral to the hemiparesis. [14]. The diagnosis of hemiplegic migraine is clinical. In cases where the diagnosis is unclear, it’s best to perform other investigations, including CT and MRI, neurovascular studies, and spinal fluid analysis to exclude other potential causes of weakness. Genetic testing is not required to make the diagnosis.

Migraine is a common symptom associated with CADASIL. The overall prevalence of migraine and migraine with aura in all CADASIL studies is 38% [15]. Some patients with CADASIL experienced atypical features including hemiplegic auras, and basilar-type migraine symptoms [15]. At this time, no studies have confirmed a correlation between NOTCH3 genotype and migraine in patients with CADASIL [4]. Proposed mechanisms by which CADASIL may cause migraines include decreased blood flow secondary to episodic ischemia, which would fit the classic vascular theory of migraine [15]. A second theory that could explain the increased prevalence of migraine in patients with CADASIL proposes that there is increased susceptibility to cortical spreading depression caused by frequent vascular damage, and increased episodes of hypoperfusion [15]. A third possibility is that the migraine area starts lower down in the brainstem area which is known to be affected in patients with CADASIL [15].

Transient ischemic attack and ischemic stroke occur readily in patients with CADASIL. Ischemic events occur in over 70% of individuals with biopsy-proven CADASIL [16]. The mean age of onset is 46.1 years and ranges from 30-66 years of age [3]. The ischemic episodes can present in various ways including the classic lacunar syndromes, pure sensory stroke, pure motor, ataxic hemiparesis, dysarthria-clumsy hand syndrome, and sensorimotor stroke [16]. The strokes tend to be recurrent and occasionally involve large arteries, but this is relatively rare. Patient education is important with a focus on modifying risk factors for stroke including hypertension, hyperlipidemia, and smoking.

Cognitive deficits are nearly as common as ischemic events in patients with CADASIL. Approximately 50% of patients with biopsy-proven CADASIL experience cognitive deficits with dementia being the most common presentation [16]. Most patients progress in a stepwise fashion over time as the number of ischemic event increases. There is a significant association between age, number of lacunar lesions, and cognitive function [17]. The most important factor determining the level of cognitive disability appears to be the number of lacunar lesions as observed on MRI [17].

Patients with CADASIL can experience a range of neuropsychiatric symptoms. Up to 30% of patients with CADASIL will experience neuropsychiatric disturbances of some kind [18]. The most common psychiatric symptoms are mood disorders with a frequency of 9% to 41% [18].

**Figure 2:**
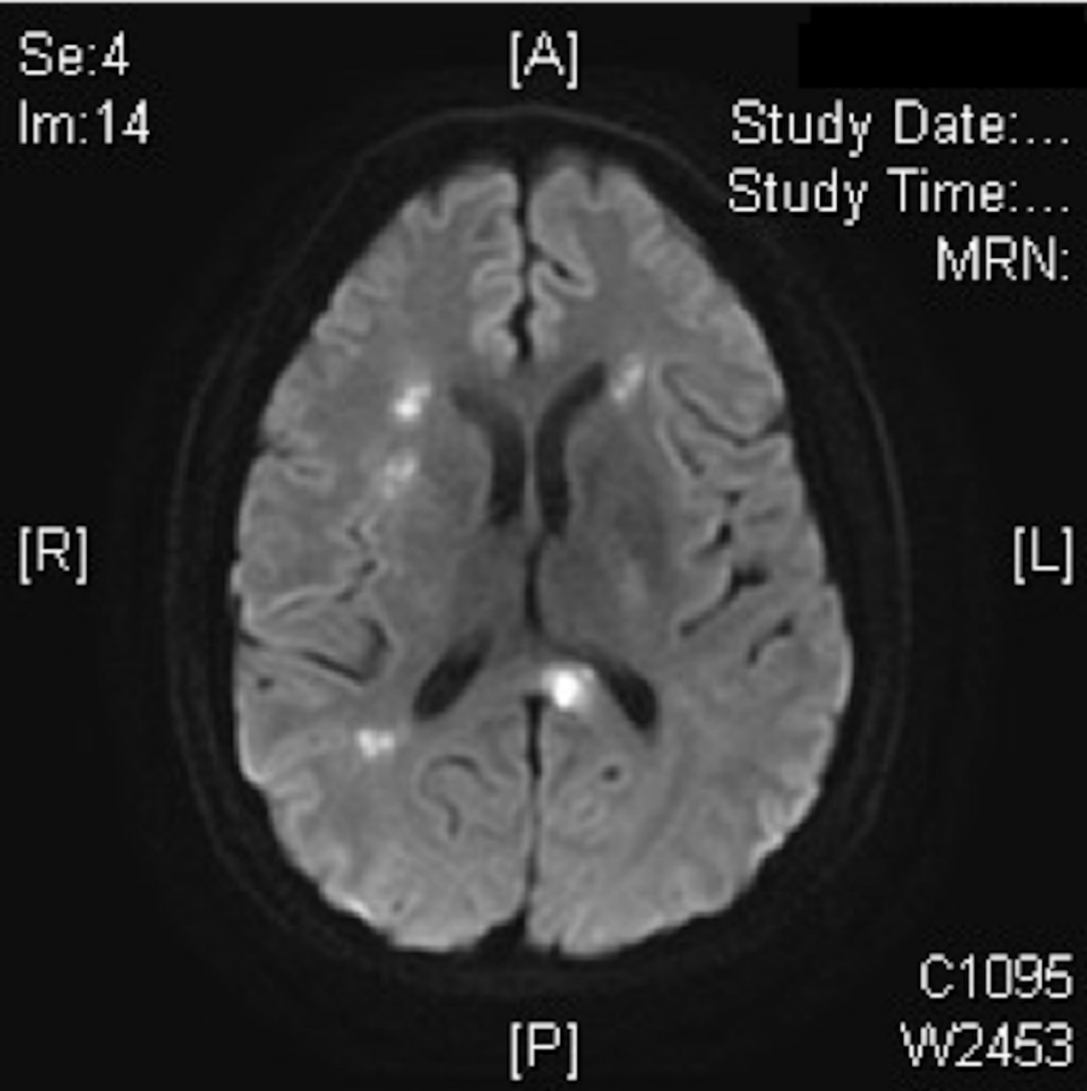
Hyperintensities on T2-weighted imaging located in the white matter, more frequent in the periventricular area

**Figure 3:**
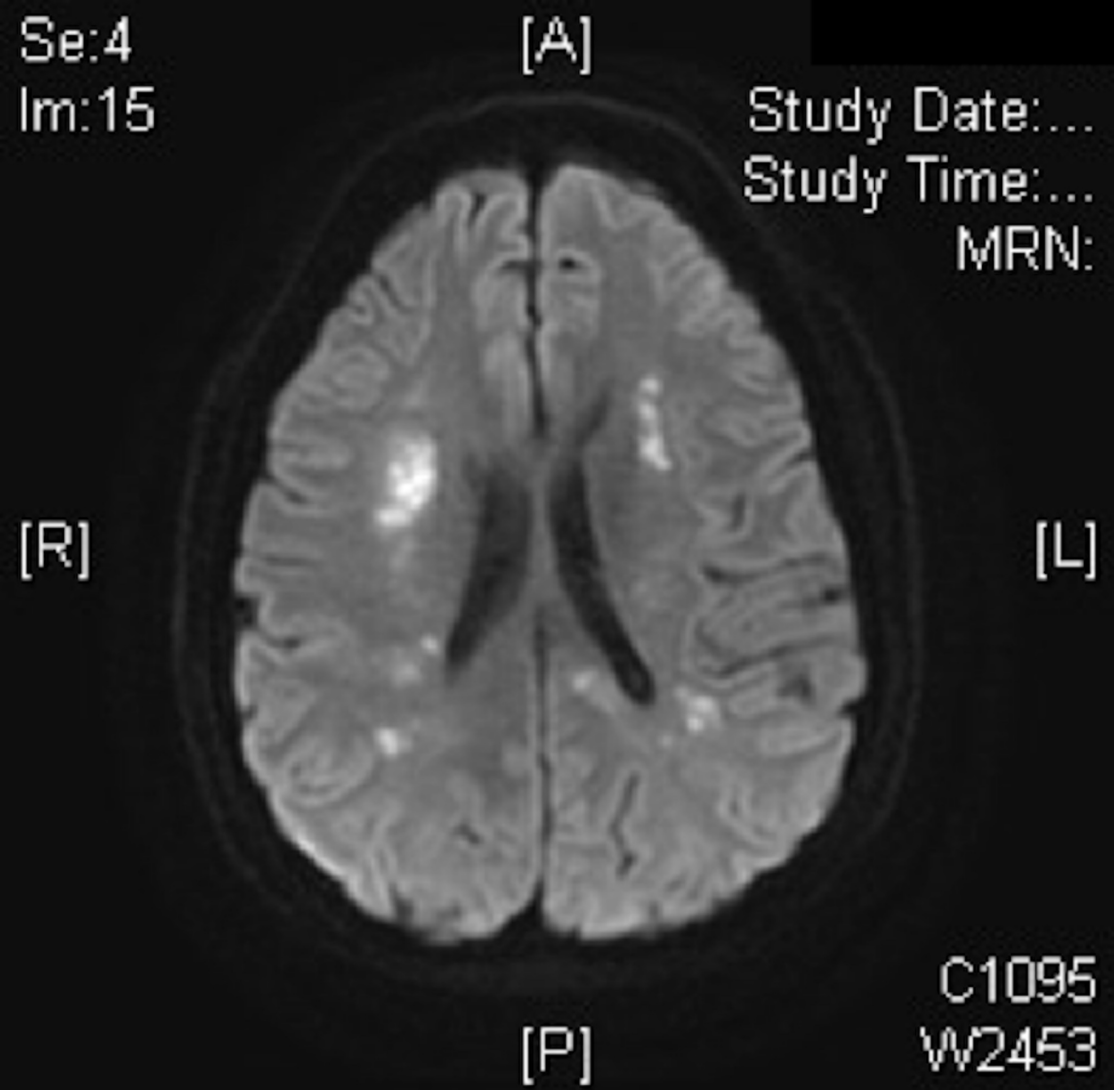
Hyperintensities on T2-weighted imaging, widespread confluent white matter hyperintensities

Chabriatet et al. found in their study of magnetic resonance imaging (MRI) on 75 patients diagnosed with CADASIL that 68 patients (90%) had hyperintensities on T2-weighted imaging located in the white matter, more frequent in periventricular (96%) and white matter (85%) than in the superficial white matter (25%) [19]. Hyperintensities also occurred in the basal ganglia (60%) and brainstem (45%) [19]. O'Sullivan et al. concluded in there study of MRI results from 20 patients diagnosed with CADASIL that temporal pole hyperintensity is a radiologic marker of CADASIL [20]. The MRI images obtained from the patient in the case corresponded well with the literature review findings as seen in Figures 2-3.

## Conclusions

This case highlights the importance of keeping an open mind when patients present with an established diagnosis. The clinical features of hemiplegic migraine have significant overlap with many of the features of CADASIL. Although CADASIL is a rare disease, had this not been part of the differential, the diagnosis might have been missed. The definitive diagnosis of CADASIL can be made using commercially available genetic testing for mutations in the NOTCH3 gene. This testing is indicated if the patient has a history suggestive of CADASIL and MRI findings that give rise to a high index of suspicion. A biopsy can also be performed to help make the diagnosis. There is no specific treatment for CADASIL at this time. The goals of treatment should be focused on symptom management and rehabilitation after ischemic injury. The most important therapy is rehabilitation since many of these patients have significant physical and cognitive disabilities that interfere with activities of daily living. The primary goal in the treatment of these patients is to keep them as functional as possible.
